# CD22 as a Target for Hematological Malignancies and Autoimmune Diseases

**DOI:** 10.3390/ijms27125406

**Published:** 2026-06-16

**Authors:** Xin Chen, Jiayi Zhang, Sizhuo Chen, Chaojun Yan, Cefan Zhou, Jingfeng Tang, Rachael Mira McLean, Zhenhuan Zhao

**Affiliations:** 1 National “111” Center for Cellular Regulation and Molecular Pharmaceutics, Key Laboratory of Fermentation Engineering (Ministry of Education), Cooperative Innovation Center of Industrial Fermentation (Ministry of Education & Hubei Province), Hubei Key Laboratory of Industrial Microbiology, School of Life and Health Sciences, Hubei University of Technology, Wuhan 430068, China; 2 Department of Public Health Dunedin, University of Otago, Dunedin 9054, New Zealand

**Keywords:** CD22, B cells, autoimmune diseases, B cell malignancies, targeted therapy

## Abstract

CD22 is a critical inhibitory coreceptor predominantly expressed on the surface of B cells, playing a pivotal role in modulating B cell receptor (BCR) signaling and maintaining immune homeostasis. Its high B cell lineage specificity, rapid internalization capacity, and signal attenuation mediated by immunoreceptor tyrosine-based inhibitory motifs (ITIMs) render it an ideal therapeutic target for B cell-related pathologies. In recent years, CD22-targeted therapeutic strategies have demonstrated significant clinical breakthroughs in the treatment of hematological malignancies and autoimmune diseases. These strategies encompass immunotoxins, radioimmunoconjugates, antibody–drug conjugates (ADCs), bispecific antibodies, and chimeric antigen receptor (CAR) T cell therapy. Notably, while monotherapies have achieved high response rates, dual-targeting approaches (e.g., CD19/CD22 CAR-T) have further mitigated the risk of antigen escape and profoundly enhanced long-term durable efficacy. This review systematically summarizes the molecular mechanisms of CD22 and the latest clinical advancements in its targeted therapies. Furthermore, we highlight the promising translational potential of CD22-targeted strategies—particularly CAR-T cell therapy—from oncology to the management of autoimmune disorders, outlining future research priorities within this rapidly evolving field.

## 1. Introduction

B cells are a core part of the adaptive immune system and play a key role in immune regulation [1,2,3]. Problems with their function are closely related to the development of many serious diseases, including B cell malignancies and autoimmune diseases.

Although CD19-targeted CAR-T therapy has achieved remarkable efficacy in certain B cell malignancies, antigen escape remains a major challenge leading to relapse [4,5,6].

Among the many molecules on the surface of B cells, CD22 has become a promising treatment target due to its unique biological features. First, its expression is highly limited to B cells, which allows targeted treatment with good cell specificity [7]. Second, CD22 is quickly taken into the cell after B cell activation [8]. CD22 undergoes continuous clathrin-mediated endocytosis without ligand stimulation, achieving 50% surface receptor internalization within 30 min [9]. Additionally, B cells harbor abundant intracellular CD22 reserves, enabling rapid replenishment of surface receptors following internalization, resulting in internalized CD22 levels reaching 2- to 3-fold the original surface expression after a single exposure [10,11]. This feature is especially useful for developing targeted drug delivery systems such as ADCs. In addition, CD22 uses its ITIM domains to suppress BCR signaling and control B cell activation [12,13]. This makes it important in treating diseases related to abnormal B cell activity.

Based on the molecular characteristics of CD22, several precise targeted treatment strategies have been developed. Monoclonal antibodies regulate B cell activation signals and mediate effector functions by binding to CD22 [14]. ADCs use CD22’s internalization feature to deliver cytotoxic drugs directly into cells [15]. Radioimmunoconjugates use CD22 targeting to bring radioactive isotopes to the tumor microenvironment, killing heterogeneous cell populations through cross-radiation effects [16]. Bispecific T-cell engagers redirect T cells by binding to both CD22 and CD3, enabling major histocompatibility complex (MHC)-independent tumor elimination [17]. CAR-T cell therapy uses engineered T cells to specifically recognize CD22 and effectively remove abnormal B cells [18]. While the clinical development of these modalities has been largely focused on B cell malignancies, the shared centrality of pathogenic B cells in certain autoimmune disorders provides a compelling rationale for their translation. The proven efficacy and safety profile of CD22-targeted therapies in eliminating B cell populations in cancer settings serve as a robust foundation for exploring their application in re-establishing immune tolerance in autoimmunity.

Currently, the clinical development of CD22-targeted therapies has primarily focused on B cell malignancies, with limited direct experience in autoimmune diseases. Nevertheless, pathogenic B cells also play a central role in certain autoimmune conditions. The established efficacy and safety of CD22-directed B cell depletion in oncology provides a rationale for exploring its potential in restoring immune tolerance in autoimmunity. In this review, we will summarize the molecular features and tumor-targeting strategies of CD22, and, where evidence permits, discuss the implications of these findings for autoimmune diseases as well as open questions for future research.

## 2. Molecular Characteristics and Function of CD22

### 2.1. Molecular Structure of CD22

CD22 is a type I transmembrane protein belonging to the sialic acid-binding immunoglobulin-type lectin (Siglec) family and is made up of three parts: an extracellular region, a transmembrane region, and an intracellular region [7,19,20]. The extracellular region contains seven immunoglobulin-like (Ig-like) domains and multiple glycosylation sites [21]. The N-terminal V-set domain (D1) specifically recognizes α2,6-linked sialic acid and is critical for ligand binding. A sialic acid-binding pocket within D1, formed by conserved arginine and aromatic residues, is essential for this specificity [22]. Domains D2–D7 are C2-set domains that contribute to ligand binding, stability and function of CD22 [21,22]. The transmembrane region consists of a hydrophobic amino acid sequence that anchors CD22 in the cell membrane, connecting the extracellular and intracellular regions [23]. The intracellular region contains seven phosphorylatable tyrosine residues (human CD22: Y762, Y773, Y783, Y807, Y822, Y842 and Y863) that are distributed among three ITIMs and one ITIM-like motif [23,24]. Among these, Y762, Y807, Y822, Y842 and Y863 become phosphorylated upon BCR activation and recruit downstream effectors: Y762 and Y822 bind Spleen tyrosine kinase (Syk); Y807 binds Grb2 and SH2-domain-containing inositol phosphatase (SHIP); and Y842 and Y863 recruit src homology 2 (SH2) domain-containing protein tyrosine phosphatase 1 (SHP-1) and SHP-2 [24]. These motifs recruit downstream signaling molecules such as SHP-1, thereby negatively regulating BCR signaling [25,26,27,28]. In addition, the intracellular region includes other regulatory sequences involved in specific signaling pathways and various cellular processes [29]. Furthermore, CD22 exhibits two ligand-binding configurations on the cell membrane: the “cis” configuration, in which CD22 binds α2,6-linked sialic acids on the same B cell, maintaining basal inhibitory activity, and the “trans” configuration, in which CD22 binds sialic acids on adjacent cells, inducing conformational changes that enhance ITIM phosphorylation and inhibitory signaling. This cis-to-trans switch is key to understanding the mechanism of CD22-targeted agents such as SM03. Of note, genetic polymorphisms in CD22 itself, as well as variations in the expression or structure of its ligand (α2,6-linked sialic acid), may affect the efficiency of this conformational switch and thereby influence therapeutic efficacy. For instance, missense mutations in CD22 may alter the binding affinity of its extracellular domain to ligands or antibodies, while genetic determinants of cell-surface sialylation levels can modulate the strength of cis-configuration binding, thereby impacting the activity of agents such as SM03 that rely on conformational switching. Therefore, considering the genetic background of CD22 and its ligand may have potential value in personalized therapy [23,30].

### 2.2. Biological Function of CD22

**Figure 1:**
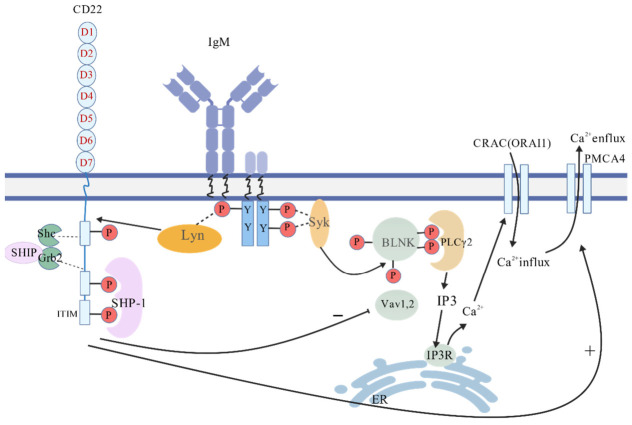
Mechanism of CD22-Mediated BCR Inhibition and Targeted Therapeutic Strategies. The extracellular region of CD22 contains seven Ig-like domains (D1–D7). Upon BCR activation, Lyn phosphorylates the ITIMs in the CD22 cytoplasmic tail (indicated by “P”). Phosphorylated ITIMs recruit SHP-1 and SHIP, which then dephosphorylate downstream signaling molecules such as BLNK and Vav1/2, thereby attenuating BCR signal transduction. Downstream signaling ultimately leads to Ca2+ release via IP3 receptors (IP3R). Multiple phosphorylation events are indicated by “P” on the respective molecules. Solid arrows (→) indicate activation or phosphorylation; dashed lines (—) indicate binding or interaction. BCR, B cell receptor; BLNK, B cell linker protein; CD22, cluster of differentiation 22; Ig, immunoglobulin; IP3, inositol 1,4,5-trisphosphate; IP3R, IP3 receptor; ITIM, immunoreceptor tyrosine-based inhibitory motif; Lyn, Src family tyrosine kinase; Vav1/2, Vav guanine nucleotide exchange factors. Created with BioGDP.com [38].

The intracellular region of CD22 contains three highly conserved ITIMs [19]. Studies show that when the BCR is activated, the Src family kinase Lyn specifically phosphorylates CD22’s ITIMs [13]. Phosphorylated ITIMs recruit the SH2 domain-containing protein tyrosine phosphatase SHP-1 as well as the SHIP. SHIP inhibits the PI3K/Akt pathway by hydrolyzing PIP3 to PI(3,4)P2; thus, like SHP-1, SHIP also negatively regulates BCR signaling, and the two molecules cooperatively amplify CD22-mediated inhibition [31,32]. In vitro experiments confirm that the CD22–SHP-1 complex effectively dephosphorylates key molecules in the BCR signaling pathway, including CD19, Syk, and BLNK, thereby weakening BCR signaling [12,33] (Figure 1). Knockout studies further support this mechanism: compared to wild-type mice, CD22-deficient B cells show stronger calcium flow responses after BCR stimulation, along with overactivation of the Erk and Akt signaling pathways [34]. In knockout mice, B-cell development is largely normal but sensitivity to autoantigens is increased [35]. In human B-cell malignancies, CD22 downregulation may relieve BCR inhibition and in some settings promote proliferation; conversely, other studies suggest that CD22 loss could slow cell cycle progression via altered SHIP/Akt signaling [36,37]. If the latter holds true, slower proliferation of CD22-negative clones would represent a potential therapeutic advantage.

### 2.3. CD22 Expression in B Cell Malignancies and Autoimmune Diseases

Beyond its role in normal B cell biology, the expression pattern of CD22 makes it a therapeutic target for various B cell-related diseases. During B-cell development, CD22 is expressed from the pre-B stage, reaches its highest levels on transitional, marginal zone, and follicular B cells, maintains moderate-to-high expression on memory B cells, and is markedly downregulated or absent on plasmablasts and plasma cells [19,39,40]. This unique expression profile provides two critical advantages for targeted therapy. First, CD22 is highly expressed on pathogenic B-cell subsets that drive both malignancies, such as precursors and blasts, and autoimmunity, such as autoreactive transitional and memory B cells [41]. Second, CD22 is absent from plasma cells, which allows selective depletion of pathogenic B cells while preserving protective humoral immunity.

In B cell malignancies, CD22 is stably and highly expressed on the majority of B-cell malignancies [41,42]. Antibody–drug conjugates developed based on this characteristic have been approved for clinical use and show definitive efficacy. Notably, some relapsed or refractory B-cell tumors exhibit downregulation of CD22 expression, which can occur through transcriptional repression, splicing alterations, or epitope masking due to glycosylation changes [43,44,45]. These mechanisms represent important routes of resistance to CD22-targeted therapies.

In autoimmunity, the rationale for CD22 targeting shares common ground with oncology. In conditions such as systemic lupus erythematosus and rheumatoid arthritis, B cells drive disease progression through autoantibody production and antigen presentation [46,47]. Autoreactive B cells consistently express CD22 on their surface, and its inhibitory function may be dysregulated, contributing to a loss of B cell tolerance [48]. Selective expression of CD22 on pathogenic B cell populations, together with its absence on plasma cells, makes CD22 an ideal target for depleting abnormal B cells while preserving protective antibodies. Although direct clinical evidence for CD22-targeted therapies in autoimmune diseases remains limited, the extensive experience and established drug development pathways from oncology provide a solid foundation and translational rationale for future expansion into autoimmunity.

Beyond expression levels, genetic variations in CD22 itself and in enzymes involved in the synthesis of its α2,6-linked sialic acid ligand may influence the efficacy of CD22-targeted therapies. For example, the Q152E polymorphism in the CD22 extracellular domain, which lies between Ig domains 2 and 3, may alter protein stability or antibody-binding epitopes [49]. This change could potentially affect recognition by monoclonal antibodies such as epratuzumab or SM03, as well as by CAR-T cells. The synonymous SNP rs34826052, also known as P768P, is associated with reduced CD22 surface expression on B cells [50]. Carriers of the A/A genotype have reduced CD22 expression, which could decrease the number of binding sites for targeted agents and thereby reduce therapeutic efficacy. Furthermore, loss-of-function mutations in SIAE, an enzyme that modifies CD22 ligands, can alter ligand structure [51,52,53]. These observations suggest that considering the genetic background of CD22 and its ligand may have potential value in the clinical application of CD22-targeted therapies.

## 3. Unmet Clinical Needs and Rationale for CD22 Targeting

Although CD19-targeted immunotherapy has shown significant success in treating hematological malignancies, drug resistance remains a major challenge. Clinical observations show that a considerable number of B-cell acute lymphoblastic leukemia (B-ALL) patients who relapse after CD19 CAR-T therapy develop tumors that are CD19-negative, or have low CD19 expression [54]. In these patients resistant to CD19-targeted therapy, CD22 expression is often preserved and remains relatively stable. This finding supports the use of CD22-targeted treatments as a promising strategy to overcome resistance to CD19 CAR-T therapy [18,55].

In the realm of autoimmune diseases, there is a parallel, unmet need for more effective and durable therapies. While B cell depletion using anti-CD20 antibodies (e.g., rituximab) has proven beneficial in diseases like RA and SLE, a significant proportion of patients exhibit inadequate response or relapse [56,57]. The mechanisms are multifactorial, including incomplete depletion of B cells, the persistence of long-lived plasma cells, and the reconstitution of autoreactive B cell clones [58]. The stable expression of CD22 on B cells, including some precursors and subsets that may be less susceptible to CD20-targeted therapy, positions CD22 as a complementary or alternative target for achieving deeper and more sustained B cell modulation in autoimmunity.

As a therapeutic target, CD22 possesses several unique advantages: its expression is highly restricted to the B cell lineage (see Section 2.3), minimizing off-target effects, and it undergoes rapid internalization upon antibody binding (see Section 2.1), making it particularly suitable for ADCs and immunotoxins. To more clearly illustrate the differences between CD22 and the established targets CD19 and CD20, the following table compares the three molecules based on key parameters (Table 1).

## 4. CD22-Targeted Therapies

### 4.1. Monoclonal Antibodies

CD22-targeted monoclonal antibodies (mAbs) exert their effects by binding to CD22 on B cells. Their mechanisms include regulating B cell activation signals, mediating antibody-dependent cellular cytotoxicity (ADCC) or antibody-dependent cellular phagocytosis (ADCP), and in some cases, inducing apoptosis [63,64,65,66]. Two anti-CD22 mAbs have advanced to late-stage clinical development: epratuzumab and SM03 (suciraslimab).

Epratuzumab is a humanized IgG1 monoclonal antibody. It was developed by transferring the complementary-determining regions (CDRs) from a mouse anti-CD22 antibody into a human IgG1 framework [67,68]. This antibody specifically recognizes an epitope on the second immunoglobulin domain of CD22 [69]. It modulates B cell function through CD22 internalization and downregulation (>80%), along with reduced expression of CD19, CD21, and CD79b [69]. It mediates ADCC but does not activate complement-dependent cytotoxicity (CDC), contributing to a favorable infusion reaction profile [70]. Two Phase III systemic lupus erythematosus (SLE) trials (EMBODY 1/2) failed to meet primary endpoints, and development has been discontinued [71].

SM03 is a chimeric anti-CD22 monoclonal antibody developed for autoimmune diseases. It exerts immunomodulatory effects by converting CD22 binding from cis-configuration, which binds to sialic acid ligands on the same B cell, to trans-configuration, which binds to ligands on adjacent cells, thereby enhancing CD22-mediated inhibition of B cell receptor signaling and preferentially targeting autoreactive B cells [72]. The mechanism of action of SM03 depends on the cis-configuration binding between CD22 and α2,6-linked sialic acids; therefore, the expression level of sialic acid ligands on the B cell surface and their glycosylation status may influence the efficacy of SM03. In a Phase II randomized controlled trial in active rheumatoid arthritis, SM03 at a cumulative dose of 3600 mg achieved a significantly higher ACR20 response rate at week 24 compared with the placebo (65.3% versus 34.0%, *p* = 0.002), with a favorable safety profile [73]. As registered on ClinicalTrials.gov (NCT04312815), the Phase III trial met its primary endpoint, but the current regulatory status remains unclear.

### 4.2. Radioimmunoconjugates

Radioimmunoconjugates are a class of targeted radiotherapeutic agents formed by conjugating anti-CD22 monoclonal antibodies with radioisotopes via stable chelators or linkers. The mechanism of action of these drugs relies on the antibody component specifically recognizing and binding to CD22 molecules on the surface of B cells. After binding, the conjugate is internalized, delivering the radionuclide into the target cell. Subsequently, the radionuclide releases high-energy beta or alpha particles, causing double-strand breaks, DNA damage, and ultimately, cell death. Additionally, their cross-fire effect on nearby CD22-negative cells helps eliminate tumor cell populations with heterogeneous antigen expression [74].

^90^Y-epratuzumab is one of the most widely studied representative drugs in this field. It uses the humanized anti-CD22 monoclonal antibody epratuzumab as a carrier, conjugated to the β-emitting isotope ^90^Y via a stable DOTA chelator. Early preclinical studies in a subcutaneous Ramos xenograft model in nude mice demonstrated its significant antitumor activity in a dose-dependent manner: a single injection of 175 μCi of ^90^Y-epratuzumab induced complete tumor regression in all mice by day 14, whereas the low-dose group exhibited only modest tumor growth delay. Notably, the study further revealed that combining ^90^Y-epratuzumab with anti-CD20 antibody veltuzumab resulted in long-term tumor-free survival in approximately 80% of mice, whereas tumors in the monotherapy groups all recurred within a few weeks, suggesting that targeting two different B cell antigens may produce synergistic effects [75].

Based on these preclinical findings, a Phase I/II clinical trial further evaluated the safety and efficacy of ^90^Y-epratuzumab in combination with veltuzumab in patients with relapsed/refractory non-Hodgkin lymphoma. The study used a fractionated dosing schedule: patients received veltuzumab on day 1, followed by ^90^Y-epratuzumab on weeks 3 and 4. Among the 15 enrolled patients, the objective response rate was 45%, with one patient with diffuse large B cell lymphoma achieving a complete response lasting more than 12 months. In terms of safety, the main dose-limiting toxicity was reversible myelosuppression; grade 3–4 thrombocytopenia or neutropenia were observed in six patients at ^90^Y-epratuzumab doses ≥9 mCi/m^2^, leading to stepwise dose reduction to 6 mCi/m^2^. No significant non-hematologic toxicities were observed, and the treatment was well tolerated [75,76].

To further improve the therapeutic index of CD22-targeted radioimmunotherapy, pre-targeted radioimmunotherapy separates antibody localization from radionuclide delivery, thereby significantly enhancing tumor-to-normal-tissue radiation ratios. A comparative study systematically evaluated the biodistribution of streptavidin–antibody conjugates targeting CD20, CD22, and HLA-DR in a pre-targeted setting. The results demonstrated that the anti-CD22 antibody (HD39) conjugated to streptavidin achieved effective tumor targeting in three B cell lymphoma xenograft models, with tumor-to-normal-tissue radiation ratios all exceeding 1, confirming the feasibility of CD22 as a target for pre-targeted radioimmunotherapy. Notably, the study found that combining all three antibody–streptavidin conjugates did not result in higher tumor uptake or more favorable tumor-to-normal-tissue ratios compared with using a single conjugate targeting the optimally expressed antigen, suggesting that clinical application should be guided by tumor antigen expression profiling to select the optimal target for individualized therapy [77].

BAY 1862864, a ^227^Th-labeled anti-CD22 antibody, completed a Phase I dose-escalation study in patients with relapsed/refractory CD22-positive B cell non-Hodgkin lymphoma (NCT02581878). Among 21 evaluable patients, the objective response rate was 25%, with one complete response and four partial responses. The most common adverse events were neutropenia and thrombocytopenia, and the maximum tolerated dose was not reached. Although α-emitters offer theoretical advantages due to their short path length and high linear energy transfer, BAY 1862864 has not advanced to further clinical development [78].

### 4.3. Antibody–Drug Conjugates (ADCs)

**Figure 2:**
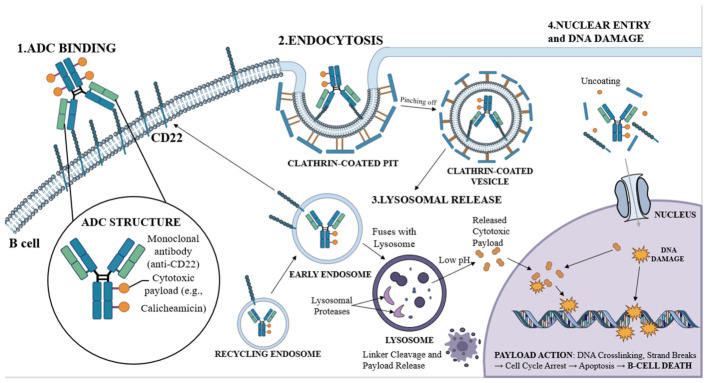
Mechanism of CD22-Targeted ADC via Clathrin-Mediated Endocytosis. The anti-CD22 monoclonal antibody is conjugated to a cytotoxic payload (e.g., calicheamicin) via a cleavable linker. Following binding to CD22 antigen on the surface of B cells, the ADC is internalized through clathrin-coated pits, a process that leads to the formation of clathrin-coated vesicles. After uncoating, the vesicles fuse with early endosomes. Under low pH conditions, early endosomes fuse with lysosomes, where lysosomal proteases cleave the linker, releasing the cytotoxic payload. The payload translocates to the nucleus and induces DNA damage through DNA crosslinking and strand breaks, leading to cell cycle arrest and apoptosis, ultimately resulting in B cell death. Arrows indicate the direction of progression.

CD22-targeted ADCs work by linking an anti-CD22 antibody to a cytotoxic drug. The ADC binds to CD22 on the B cell surface, followed by clathrin-mediated endocytosis, intracellular trafficking to the lysosome, linker cleavage, and release of the cytotoxic payload, which then induces DNA damage, cell cycle arrest, and apoptosis (Figure 2).

Inotuzumab ozogamicin is a CD22-targeted ADC consisting of a humanized anti-CD22 antibody linked to the potent toxin calicheamicin. The FDA approved it in 2017 for treating R/R B-ALL [79]. It is the only approved ADC that targets CD22. The antibody part binds to CD22 on B cells. The drug is then taken into the cell. Inside, the acidic environment releases the toxin. This toxin damages the tumor cell’s DNA, causing cell death. This targeted method reduces the widespread toxicity of standard chemotherapy.

The INO-VATE study (NCT01564784) was a key Phase III clinical trial. It evaluated inotuzumab ozogamicin for relapsed or refractory B-ALL. This international multicenter study compared inotuzumab ozogamicin with standard chemotherapy. Results showed that the complete response rate was 80.7% in the inotuzumab ozogamicin group. This was much higher than the 29.4% rate in the chemotherapy group. The median progression-free survival was also longer: 5.0 months vs. 1.8 months [80]. Additionally, 41% of patients in the inotuzumab ozogamicin group later received a successful stem cell transplant. In the chemotherapy group, only 11% did. Although there was an 11% risk of liver sinus blockage, the study confirmed the strong efficacy of CD22-targeted therapy in B-ALL [80].

Inotuzumab ozogamicin therapy is associated with a risk of sinusoidal obstruction syndrome (SOS), a severe liver toxicity characterized by endothelial injury. In the INO-VATE trial, the overall SOS rate was 13–14% in the inotuzumab ozogamicin arm versus 2–3% in the standard-of-care arm [81,82]. Among patients who proceeded to allogeneic hematopoietic stem cell transplantation after inotuzumab ozogamicin, the SOS rate was 22% compared with 3% in those receiving standard chemotherapy [82]. Mechanistically, non-specific uptake of calicheamicin by hepatic sinusoidal endothelial cells leads to endothelial damage and sinusoidal obstruction; the absence of CD22 expression in normal liver indicates that this toxicity is payload-driven rather than on-target. Multivariate analyses identified conditioning regimens containing two alkylating agents and pre-transplant bilirubin levels ≥ upper limit of normal as independent risk factors for SOS [82].

As a preliminary speculation based on the above mechanisms, we propose a hypothesis that remains to be tested: SOS may be related to direct, non-specific calicheamicin toxicity to hepatic sinusoidal endothelial cells [83]. The observation in INO-VATE that SOS risk correlates with the number of treatment cycles, together with clinical data showing that fractionated/reduced-dose regimens lower SOS rates to approximately 2%, suggests that reducing systemic calicheamicin exposure might lower SOS risk [84]. Furthermore, a more distant speculation is that developing payloads or linkers with different profiles of hepatic sinusoidal endothelial protection could theoretically reduce SOS without compromising antitumor efficacy, but this awaits future investigation.

Other CD22-targeting ADCs that have entered clinical development include pinatuzumab vedotin, ADCT-602, AZD4512, and TRPH-222. However, the development of pinatuzumab vedotin and ADCT-602 has been terminated. AZD4512 is currently being evaluated in Phase I/II trials for B-ALL and B-NHL. TRPH-222 completed a Phase I study in B cell NHL, but its further development status remains unclear.

### 4.4. Immunotoxins

CD22-targeted immunotoxins are biologic agents created by linking CD22-specific antibody fragments to potent protein toxins. These drugs use the antibody portion to specifically recognize CD22 on B cells. Through receptor-mediated internalization, the toxin is delivered into the cell, where it irreversibly inhibits protein synthesis, leading to apoptosis of the target cells [85].

Moxetumomab pasudotox (MP) is a recombinant immunotoxin that targets CD22. It is composed of an anti-CD22 single-chain antibody fragment fused to a 38 kDa fragment of Pseudomonas exotoxin A, called PE38, which is expressed in *E. coli* [85]. The drug received FDA approval in September 2018 for the treatment of relapsed or refractory hairy cell leukemia (HCL) in adults who have received at least two prior systemic therapies, including a purine nucleoside analog.

The Phase III pivotal study (CD-ON-CAT8015-1053) enrolled 80 patients, who received MP at a dose of 40 μg/kg on days 1, 3, and 5 of each 28-day cycle for up to six cycles. The combined complete and partial response rate was 79%, and 80% of patients achieved hematologic remission. Among responders, 85% tested negative for minimal residual disease by immunohistochemistry [86].

Common adverse events associated with MP included infusion-related reactions (≤20%), diarrhea (≤50%), nausea, edema, headache, anemia, fever, as well as renal toxicity and electrolyte disturbances. Serious adverse events included capillary leak syndrome and hemolytic uremic syndrome. We hypothesize that the development of capillary leak syndrome may be related to toxin immunogenicity and dose intensity; de-immunization or fractionated dosing strategies might reduce this risk.

Immunogenicity was common in the Phase III study, with 59% of patients testing positive for anti-drug antibodies; of these, 95.7% had detectable neutralizing antibodies [86]. In November 2022, the manufacturer notified the FDA of its decision to discontinue MP, citing “very low clinical uptake”. The discontinuation took effect in August 2023. The decision was not based on concerns regarding the safety or efficacy of the drug but rather on factors such as the complexity of administration, the need for patient monitoring, and potential prophylactic toxicity, which may have limited its clinical use.

Other CD22-targeting immunotoxins that have entered clinical development include DT2219ARL and Combotox. DT2219ARL is a bispecific immunotoxin targeting both CD19 and CD22, composed of anti-CD19 and anti-CD22 scFv fragments linked to a truncated diphtheria toxin. Combotox is a 1:1 mixture of two ricin A chain-based immunotoxins targeting CD19 and CD22, which has been evaluated in Phase I trials for refractory B-ALL, showing modest activity but dose-limiting vascular leak syndrome [87,88]. Neither agent has advanced to Phase III development.

### 4.5. Bispecific Antibodies

Bispecific antibodies are engineered antibody molecules that can bind to two different antigens or two different parts of the same antigen at the same time. For treating B cell cancers, most CD22-targeted bispecific antibodies use a CD22/CD3 design. One end binds to CD22 on B cells, and the other end binds to CD3 on T cells. This creates an artificial immune synapse between T cells and tumor cells [17]. This design bypasses the need for MHC recognition, directly activating the patient’s own T cells and directing them to kill CD22-positive tumor cells [17]. This method is called T-cell engager therapy. In theory, this approach can kill tumor cells even with low CD22 expression. It also helps prevent immune escape caused by loss or reduction in a single antigen.

Preclinical studies have shown that CD22/CD3 bispecific antibodies induce T-cell-mediated cytotoxicity in a dose-dependent and effector-to-target ratio-dependent manner in vitro, and inhibit tumor growth in an acute lymphoblastic leukemia xenograft mouse model [17].

Currently, several CD22-targeting bispecific antibodies have entered clinical evaluation. JNJ-75348780, a humanized CD3/CD22 bispecific antibody administered subcutaneously, was investigated in a first-in-human Phase I trial (NCT04540796) for relapsed/refractory B-cell non-Hodgkin lymphoma and chronic lymphocytic leukemia. REGN5837, a CD22/CD28 bispecific antibody designed to provide co-stimulatory signaling (Signal 2), is being evaluated in combination with the CD20/CD3 bispecific antibody odronextamab. Preclinical studies demonstrated that REGN5837 has limited activity as a monotherapy but significantly enhances T cell activation and anti-tumor activity when combined with odronextamab. This combination is currently under investigation in a Phase I clinical trial for patients with relapsed/refractory aggressive B-cell non-Hodgkin lymphoma (NCT05685173).

### 4.6. CD22 CAR-T Cell Therapy

Since the first CAR-T product (CD19-targeted Kymriah) was approved in 2017, CAR-T therapy has shown revolutionary efficacy in B cell malignancies. In children and adults with R/R ALL, complete remission (CR) rates have been reported in approximately 60 to 90% of patients receiving CD19 CAR-T cell therapy [89,90,91,92]. However, although initial remission rates exceeding 80% have been observed across multiple studies involving both FDA-approved and investigational CD19 CAR-T cell products, 36% to 57% of patients who achieve complete remission relapse within one year, and ultimately only approximately 40% to 50% of patients maintain durable remission [93]. The emergence of CD19-negative relapse and the limited persistence of CAR-T cells are the main challenges facing current CD19 CAR-T cell therapy.

CD22 remains stably expressed in relapsed patients, and early clinical trials show a 40–70% secondary response rate, making it one of the most promising alternative targets [18,94].

CD22 CAR-T therapy has a core design with three parts: an anti-CD22 single-chain antibody (like m971) for antigen recognition, a CD28 or 4-1BB co-stimulatory domain to control T cell activation and persistence, and a CD3ζ chain to deliver the activation signal [18].

A Phase I clinical trial conducted by the National Cancer Institute (NCT02315612) evaluated for the first time the efficacy of CD22 CAR-T (m971-BBz) in patients with relapsed or refractory B-ALL. The study used a 3 + 3 dose-escalation design, with patients receiving cyclophosphamide/fludarabine (Cy/Flu) lymphodepletion prior to CD22 CAR-T infusion [18]. A total of 21 patients were enrolled, with a median age of 19 years. Among them, 17 had previously received CD19-directed therapy, and 10 had dim or negative CD19 expression at enrollment. Twelve patients (57%) achieved complete remission, of whom 9 were minimal residual disease negative. Among the 15 patients who received the effective dose level (≥1 × 10^6^ cells/kg), 11 (73%) achieved complete remission. The median duration of remission was 6 months. Sixteen of the 21 patients experienced cytokine release syndrome (76%), mostly grade 1–2. No dose-limiting toxicities were observed at the 1 × 10^6^ cells/kg dose level, which was selected as the recommended Phase II dose. All patients who achieved complete remission developed B cell aplasia, and no irreversible neurotoxicity was observed. Among the eight patients who relapsed after achieving remission, seven showed downregulation of CD22 expression. Further analysis revealed no mutations in the CD22 gene or reductions in mRNA levels, suggesting that downregulation of CD22 protein expression may be the primary mechanism by which leukemia cells escape CD22 CAR-T killing [18].

To optimize the efficacy and safety of CD22 CAR-T, a multicenter Phase I clinical trial (NCT04088890) conducted dose-finding studies in patients with B-ALL and Large B-cell lymphoma (LBCL), respectively [95,96].

In the B-ALL cohort, the National Cancer Institute team evaluated three dose levels (3 × 10^5^, 1 × 10^6^, and 3 × 10^6^ cells/kg) in 58 children and young adults. The complete remission rate was 70%, with a median relapse-free survival of 6.0 months. Although the introduction of CD4/CD8 T cell selection improved manufacturing success rates, it also significantly increased inflammatory toxicities such as HLH/MAS-like syndrome, leading to a final recommended dose reduction to 3 × 10^5^ cells/kg without compromising efficacy.

In the LBCL cohort, the Stanford University team evaluated two dose levels (1 × 10^6^ and 3 × 10^6^ cells/kg) in 38 adult patients who had relapsed after prior CD19 CAR-T therapy. The maximum tolerated dose was determined to be 1 × 10^6^ cells/kg, at which no dose-limiting toxicities or grade ≥3 cytokine release syndrome or immune effector cell-associated neurotoxicity syndrome were observed. The objective response rate was 68%, the complete remission rate was 53%, and the 2-year overall survival rate was 52%, Together, these two studies demonstrate that CD22 CAR-T is efficacious across different B cell malignancies, although toxicities and optimal dosing vary by disease type and manufacturing process.

**Figure 3:**
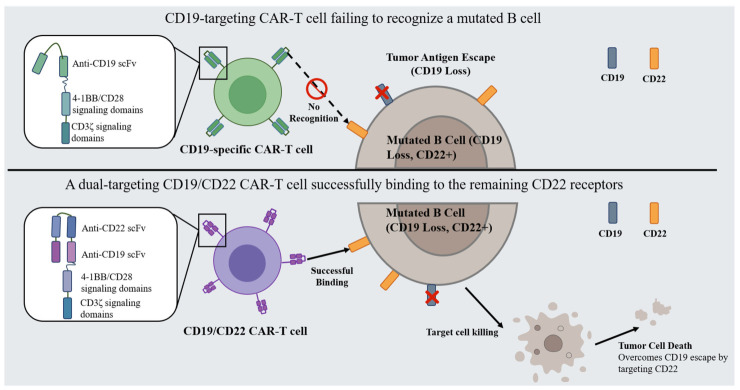
Schematic illustration of CD19 antigen escape and its mitigation by CD19/CD22 dual-targeting CAR-T cells. A CD19-specific CAR-T cell fails to recognize a mutated B cell that has lost CD19 expression (CD19−, CD22+), leading to tumor antigen escape and subsequent relapse. In contrast, a dual-targeting CD19/CD22 CAR-T cell successfully binds to the remaining CD22 receptors on the same mutated B cell, triggering target cell killing and overcoming CD19 escape-mediated resistance. This dual-targeting strategy enables more comprehensive clearance of B cell malignancies. Red cross (x) indicates loss.

In recent years, continued progress has been made in the field of CD22 CAR-T. A ten-year follow-up study summarizing long-term data from 78 patients with B-ALL who received the same product reported a complete remission rate of 70.1% and a median overall survival of 13.6 months, further confirming the durable value of CD22 CAR-T. To further improve efficacy, a “sandwich” strategy combining sequential CD22/CD19 CAR-T with autologous stem cell transplantation was explored, achieving a 2-year overall survival rate of 97% and a 2-year leukemia-free survival rate of 72% in 37 patients. In addition, dual-targeting CAR-T (CD19/CD22) in 35 patients showed a median overall survival of 21.5 months, with a cytokine release syndrome rate of only 37.1% [97]. As illustrated in Figure 3, CD19-specific CAR-T cells fail to recognize CD19-negative tumor cells, whereas CD19/CD22 dual-targeting CAR-T cells overcome this resistance by engaging the residual CD22 antigen. To address antigen escape, a growing number of ongoing clinical trials are now focusing on dual- or multi-targeting CAR-T constructs. These advances provide new directions for further optimization of CD22-targeted therapy.

The established efficacy of CD22-directed CAR-T therapy in B cell malignancies, particularly in overcoming CD19 antigen escape, provides a robust translational rationale for its application in autoimmune diseases. This potential stems from a shared pathogenic mechanism: dysregulated B cells are central drivers of conditions such as SLE and rheumatoid arthritis (RA) [98,99,100]. Consequently, strategies capable of profound B cell depletion in oncology may be repurposed to modulate the autoreactive immune system, representing a shift from tumor eradication to immune system regulation.

Compared to existing B-cell-targeted therapies, CAR-T offers distinct potential advantages. While conventional anti-CD20 monoclonal antibodies are effective, they face limitations such as incomplete responses, high relapse rates, and an inability to eliminate all B cell subsets [56]. In contrast, CAR-T therapy can achieve deep and sustained B cell depletion, offering the possibility of inducing long-term clinical remission [101,102,103,104].

This concept has received preliminary clinical validation. Recent breakthroughs with CD19-targeted autologous CAR-T therapy in patients with severe, refractory SLE have demonstrated deep and sustained clinical and serological remission, enabling discontinuation of immunosuppressants in some cases [101,102,103]. This proof of concept confirms that B-cell-directed CAR-T strategies are both feasible and highly effective in autoimmunity. Given the well-documented risk of antigen escape in oncology, employing CD22 as a primary or complementary target for autoimmune CAR-T therapy is a strategically sound approach, potentially enabling more comprehensive and reliable B cell clearance [95,105].

Recent advances in cellular therapy for autoimmunity extend beyond autologous CAR-T, pointing toward directions with improved accessibility and safety [106]. A clinical study published in *The Lancet* in November 2025 by a team from Shanghai’s Changhai Hospital reported on the efficacy and safety of allogeneic CD19 CAR-NK cells (KN5501) in 18 patients with refractory, moderate-to-severe SLE. After over one year of follow-up, this therapy resulted in complete remission or low disease activity in 67% of patients, with no observed relapses. The treatment exhibited a favorable safety profile, with only one transient fever reported and no incidence of severe cytokine release syndrome, neurotoxicity, or infection [107].

Building on this precedent, a key future direction for CD22-targeted cellular therapies lies in developing allogeneic products based on the CD22 antigen (e.g., CAR-NK or universal CAR-T). This approach would not only leverage the advantage of CD22 in addressing antigen escape, but also incorporate the progress in accessibility, safety, and scalable manufacturing offered by allogeneic platforms.

CD22 CAR-T therapy represents a promising avenue for research. Early proof of concept and recent technological breakthroughs jointly indicate that the future lies in integrating optimal targets with optimized platforms, ultimately offering new therapeutic options for patients with severe autoimmune diseases.

## 5. Summary and Outlook

**Figure 4:**
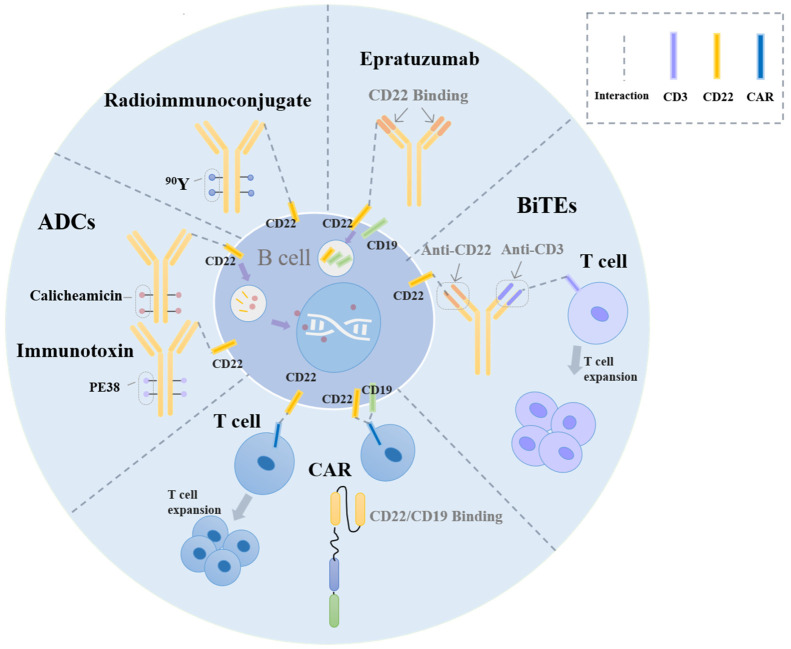
Overview of CD22-Targeted Therapeutic Strategies for B Cell Malignancies. This figure illustrates multiple CD22-targeted therapeutic approaches: monoclonal antibodies (e.g., Epratuzumab) target B cells via mechanisms such as ADCC/CDC; ADCs deliver cytotoxic agents (e.g., calicheamicin) to induce DNA damage; immunotoxins (ITs) deliver protein toxins to inhibit protein synthesis; radioimmunoconjugates (RITs) utilize radionuclides to eradicate tumor microenvironments; bispecific T-cell engagers (BiTEs) simultaneously bind CD22 and CD3 to activate T cells; and CAR-T cells are engineered to specifically recognize CD22 and mediate cytotoxicity. The figure demonstrates the mechanisms of action of these therapies, providing multidimensional strategies for treating B cell malignancies. ADC, antibody–drug conjugate; BiTE, bispecific T-cell engager; CAR-T, chimeric antigen receptor T-cell; IT, immunotoxin; RIT, radioimmunotherapy.

CD22 has emerged as a versatile therapeutic target due to its restricted B cell expression profile, efficient internalization capacity, and ability to negatively regulate B cell receptor signaling through its ITIM domains. Various targeting strategies developed against CD22 include monoclonal antibodies, antibody–drug conjugates, immunotoxins, bispecific antibodies, and CAR-T cells. These agents have demonstrated clinical activity in B cell malignancies and have recently shown promise in autoimmune diseases (Figure 4 and Table 2).

Despite these advances, several challenges remain. First, the early development of CD22-targeted antibodies faces two major technical hurdles. On one hand, the heavy glycosylation of CD22 epitopes severely impairs antibody binding, rendering the generation of high-affinity antibodies highly challenging. On the other hand, the rapid internalization that occurs upon antibody binding complicates the establishment of suitable assays to monitor changes in drug bioactivity during both in vitro and in vivo studies. Second, CD22 downregulation following CD22 CAR-T therapy represents a major mechanism of resistance, limiting durable responses. Third, the toxicity profiles of CD22-targeted agents, such as sinusoidal obstruction syndrome associated with inotuzumab ozogamicin or capillary leak syndrome linked to immunotoxins, require careful clinical management. Fourth, the logistical complexity and high cost of autologous CAR-T manufacturing hinder widespread accessibility, particularly in autoimmune indications where treatment paradigms are less established.

Looking forward, three directions warrant particular attention. First, multi-targeting strategies such as CD19 and CD22 dual-targeting CAR-T are being increasingly explored to prevent antigen escape, with several ongoing clinical trials expected to clarify their optimal design and positioning. Second, novel delivery platforms including messenger RNA lipid nanoparticle-based in vivo T cell reprogramming and allogeneic CAR-NK cells promise to overcome the manufacturing and accessibility barriers of current autologous products [108]. Third, the successful application of CD19-targeted CAR-T in refractory lupus provides a strong rationale for extending CD22-targeted cellular therapies to a broader spectrum of autoimmune diseases, potentially offering a curative approach for patients who fail conventional immunosuppression.

Ultimately, the goal of CD22-targeted therapy extends beyond eliminating pathogenic B cells to restoring long-term immune homeostasis. Translating the lessons learned from oncology into autoimmune settings will require careful optimization of safety, efficacy, and accessibility. With continued innovation, CD22-targeted platforms are poised to become transformative tools in both cancer immunotherapy and autoimmune disease modification.

## Figures and Tables

**Table 1:** Comparison of CD22, CD19, and CD20 as therapeutic targets.

	CD22	CD19	CD20
Broad expression	Pre-B to memory, not plasma cells [59]	Pro-B through plasma cell differentiation [60]	Pre-B to memory, not plasma cells [61]
Internalization rate	Fast [62]	Moderate	Slow/none
Signaling function	Inhibitory	Activating	Modulates calcium flux
Internalization upon antibody binding	Yes, rapid and efficient	Yes, partial	Minimal/none
Current clinical agents	Inotuzumab ozogamicin, epratuzumab, anti-CD22 CAR-T	Tisagenlecleucel, blinatumomab, Loncastuximab tesirine	Rituximab, Obinutuzumab, Ofatumumab

**Table 2:** Representative clinical trials using CD22-targeted therapy for B cell malignancies and autoimmune diseases (registered at ClinicalTrials.gov).

Category	Agents	Indication	Mechanism	Key Efficacy	Major Toxicities	Clinical Status	Advantages	Limitations
mAbs	Epratuzumab	SLE	Anti-CD22 naked mAb	SLE Phase IIb positive; Phase III failed	Infusion reactions, headache	Discontinued (NCT00624351)	Non-depleting; low infection risk	No approved indication; SLE Phase III failure
	SM03	RA	Anti-CD22 naked mAb	RA Phase III met ACR20 primary endpoint	Well-tolerated, rare SAEs	Phase III completed (NCT04312815)	First anti-CD22 mAb with positive Phase III in autoimmunity	Limited long-term data; only evaluated in RA
RIT	^90^Y-Epratuzumab	NHL	Anti-CD22 mAb conjugated with β-emitter ^90^Y for targeted radiation	Phase I/II: ORR 45% (in combination with veltuzumab) in NHL	Thrombocytopenia, neutropenia, long-term myelotoxicity	Discontinued	Strong cytotoxicity; suitable for bulky tumors	Significant toxicity; no further development; not approved
ADCs	Inotuzumab ozogamicin	B-ALL	Anti-CD22 mAb + calicheamicin	B-ALL CR/CRi ~81%	SOS/VOD, myelosuppression, infection	FDA approved (NCT01564784)	Highly effective in R/R B-ALL; only approved CD22 ADC	Black box warning for SOS; limited activity in NHL
IT	Moxetumomab pasudotox	HCL	Anti-CD22 dsFv + PE38	HCL CR 41–55%	HUS, capillary leak syndrome	FDA approved (NCT01829711)	Durable responses in HCL	High toxicity; anti-drug antibodies
BiTEs	CD22/CD3 bispecific Ab	NHL	Redirects T cells to kill CD22^+^ B cells	Early activity in NHL	CRS, neutropenia	Phase I (NCT04540796)	Potent T cell engagement	Toxicity; limited mature clinical data
CAR-T	CD19/CD22 CAR-T	B-ALL	Dual-targeting to prevent antigen loss	R/R B-ALL CR 70–90%	CRS, ICANS, cytopenia	Phase II/III (NCT03448393)	Reduces CD19-negative relapse; superior to single CD19 CAR-T	Complex manufacturing; high cost
	CD22 CAR-T	B-ALL	Single-target CD22 CAR-T	B-ALL CR ~70%	CRS, infection	Phase I/II (NCT02315612)	Option for CD19-negative patients	High risk of CD22 loss and relapse

## Data Availability

No new data were created or analyzed in this study. Data sharing is not applicable to this article.

## Abbreviations

The following abbreviations are used in this manuscript:

ITIM	Inhibitory Motif
BCR	B cell Receptor
ADC	Antibody–Drug Conjugate
CAR	Chimeric Antigen Receptor
MHC	Major Histocompatibility Complex
Siglec	Sialic Acid-Binding Immunoglobulin-Type Lectin
Ig-like	Immunoglobulin-Like
SHP-1	Src Homology 2 (SH2) Domain-Containing Protein Tyrosine Phosphatase 1
SHIP	SH2-Domain-Containing Inositol Phosphatase
B-ALL	B-cell Acute Lymphoblastic Leukemia
mAbs	Monoclonal Antibodies
ADCC	Antibody-Dependent Cellular Cytotoxicity
ADCP	Antibody-Dependent Cellular Phagocytosis
CDR	Complementary-Determining Region
CDC	Complement-Dependent Cytotoxicity
SOS	Sinusoidal Obstruction Syndrome
MP	Moxetumomab Pasudotox
HCL	Hairy Cell Leukemia
CR	Complete Remission
SLE	Systemic Lupus Erythematosus
RA	Rheumatoid Arthritis
